# Semantic Changepoint Detection for Finding Potentially Novel Research Publications

**Published:** 2021

**Authors:** Bhavish Dinakar, Mayla R. Boguslav, Carsten Görg, Deendayal Dinakarpandian

**Affiliations:** Department of Chemical and Biomolecular Engineering, University of California, Berkeley Berkeley, CA 94720; Computational Bioscience Program, University of Colorado Anschutz Medical Campus Aurora, CO 80045; Department of Biostatistics and Informatics, University of Colorado Anschutz Medical Campus Aurora, CO 80045; Center for Biomedical Informatics Research, Stanford University Stanford, CA 94305

**Keywords:** Changepoint, Semantic, Novel research paper, Literature search

## Abstract

How has the focus of research papers on a given disease changed over time? Identifying the papers at the cusps of change can help highlight the emergence of a new topic or a change in the direction of research. We present a generally applicable unsupervised approach to this question based on semantic changepoints within a given collection of research papers. We illustrate the approach by a range of examples based on a nascent corpus of literature on COVID-19 as well as subsets of papers from PubMed on the World Health Organization list of neglected tropical diseases. The software is freely available at: https://github.com/pdddinakar/SemanticChangepointDetection.

## 1. Introduction

There are several possible motivations behind a literature search. These range from finding the answer to a highly specific question to writing a general review of a topic. One of the motivations for a literature review might be to select a topic for research, where one may choose to perform research in a well-established area, pick an emerging area or aspire to be a pioneer in uncharted territory. Another possible motivation might be for a funding agency to keep track of emerging areas of research that might merit funding in the near future. Yet another motivation might be to keep track of new insights or technologies that address an acute health need such as a pandemic or diseases that are hard to treat effectively.

What if it were possible to identify papers that strayed from the mainstream? While many of these might end up as blind alleys, a subset of these might turn out to be harbingers of innovative, influential, or impactful directions in research. A few of the potential approaches to identify outliers, first-to-report, or first-in-field papers are topic modeling^1^, clustering^2^, trend analysis^3,4^, citation network analysis^5^, and machine learning approaches for predicting high impact papers. We present a set of strategies from changepoint analysis and text embedding to address two questions. Which are the papers in a research area that are substantially different from previous work? Which papers are part of a related cluster that is substantially different from previous work? We use infectious diseases from two different time scales to illustrate the approach - COVID-19^6^ over a temporal resolution of weeks, and leprosy^7^, considered a neglected tropical disease by the World Health Organization. We begin with a description of the methods used, followed by results and discussion, and end with an acknowledgment of the limitations and future work to address them.

## 2. Methods

**Figure 1.**
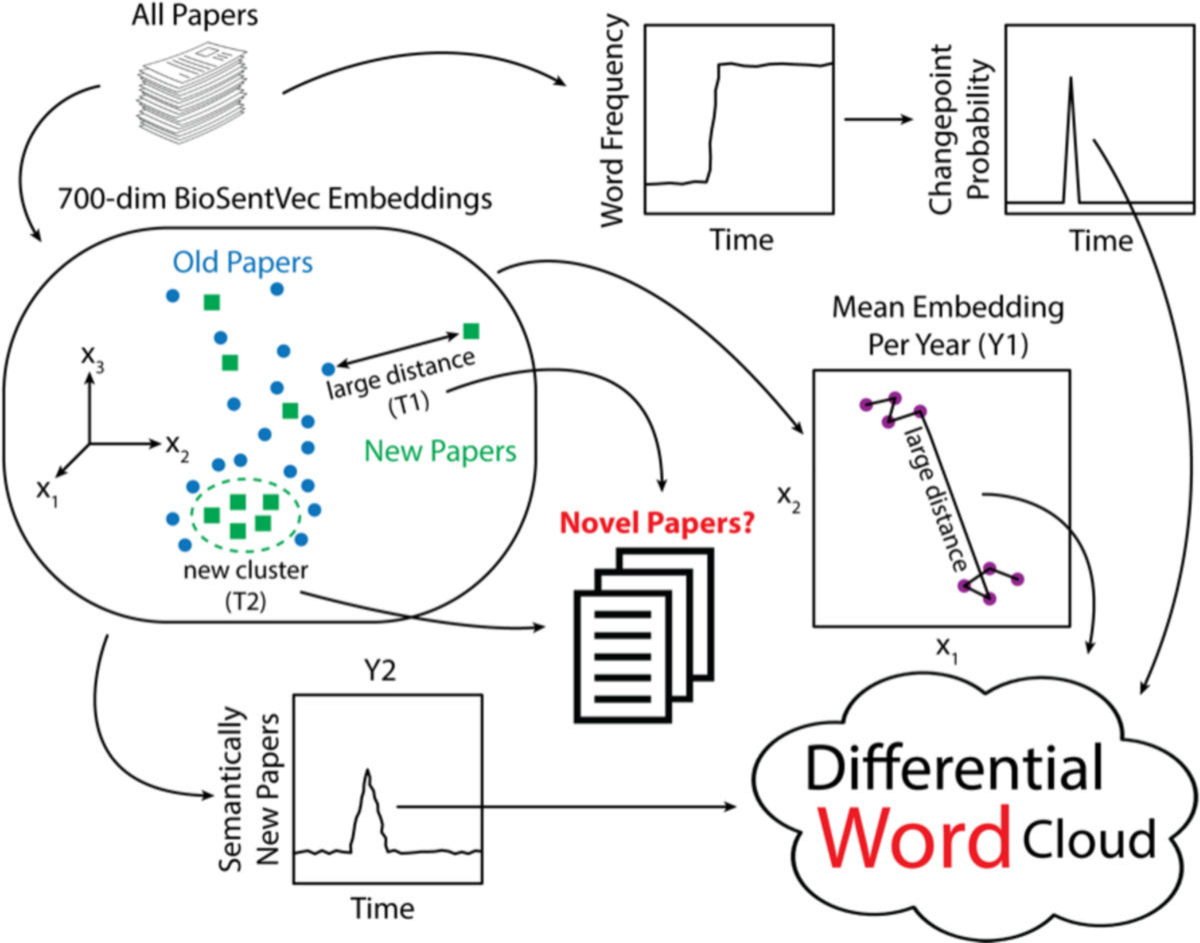
Overview of semantic changepoint analysis using word frequency-based and embedding space-based strategies (T1, T2, Y1, Y2).

The overall summary of the methodology is shown in Fig. 1. Briefly, titles or abstracts from a collection are either embedded as a vector or represented in terms of word frequency distributions. Temporal changes in these representations of titles or abstracts are detected by approaches described below. Papers or terms corresponding to the temporal changes are marked as potentially novel for the corresponding time period.

### 2.1 Data collection and general procedures

COVID papers were downloaded from the COVID-19 SARS-CoV-2 Preprints available from bioRxiv in JSON format on 7/31/2020.^8^ The title, upload date, and abstract were retrieved for each paper published in 2020, yielding a total of 7151 analyzed papers.

Leprosy papers were retrieved from the National Center for Biotechnology Information E-Utilities API. A list of UIDs for papers where the term “leprosy” appears in the title were retrieved using the ESearch method, and the title, abstract, and publication date for each UID were retrieved with the EFetch method. We only analyzed papers for which the title, abstract, and publication date were available. We also only considered papers published between 1980 and 2019 due to the low number of annual papers (less than 50 per year) published before 1980, yielding a total of 5068 analyzed papers. Plots were generated using the matplotlib package^9^.

### 2.2 Title and abstract entropies

We used the scikit-learn^10^ CountVectorizer tool to convert each title to a Bag-of-Words representation. We calculated the probability of each word in a year using Eq. (1).

(1) $$p_{\text{word,year}}=\frac{\text{frequency}\;\text{of}\;\text{word}\;\text{in}\;\text{year}}{\text{total}\;\text{frequency}\;\text{of}\;\text{all}\;\text{words}\;\text{in}\;\text{year}}$$

The yearly entropy of word proportions in titles (or alternatively, in abstracts) was calculated by Shannon entropy, which is a popular measure of information content or variability of a distribution.

(2) $$S_{\text{year}}=-\sum_{\text{word}}p_{\text{word,year}}\log_{2}\left(p_{\text{word,year}}\right)$$

### 2.3 Bayesian changepoint analysis

Changepoint detection aims to identify the point at which the probability distribution of a sequential variable changes. Changepoint analysis was conducting using the bayesian-changepoint-detection (bcp) Python package.^11–13^ The advantage of the bcp method is that it also provides a probability of there being a changepoint at a given time point. We performed changepoint detection for each word in the Bag-of-Words model of paper titles to analyze the word frequency per title vs time (year for Leprosy, 2020 week number for COVID). Abstract changepoints were calculated in the same manner, except using abstracts as input to the Bag-of-Words model instead of titles.

### 2.4 Differential word clouds

Differential word clouds are visual depictions of changes in research paper titles between two years, denoted Year A and Year B. Two groups are selected from the paper titles: Group A contains the titles of all papers published in or before Year A, and Group B contains the titles of all papers published in Year B. The Bag-of-Words model is applied to each group to determine the frequency per title of each word, and stop-words appearing in the NLTK stop-words set are removed.^14^ Word clouds were created using the Wordcloud Python package,^15^ with weights for each word corresponding to the difference in frequency per title between Group B and Group A. Positive differences (increases in word frequency) are colored black, and negative differences (decreases in word frequency) are colored red.

### 2.5 Title and abstract embeddings

Titles were first pre-processed by converting all words to lowercase and removing punctuation and stop-words found in the NLTK punctuation and stop-word sets. The processed titles were then converted into 700-dimensional vectors using the BioSentVec model^16^. Abstracts were embedded in the same manner, except using the abstract as the input instead of the title. The embeddings were visualized using Principal Component Analysis as implemented by Scikit-Learn.

### 2.6 Semantic novelty

We use the following approaches to detect potentially novel papers or subtopics in one temporal window (or subcollection) with respect to another. For instance, one may compare papers in 2020 with all preceding years (novel compared to entire research legacy). Alternatively, one may compare papers published in 2020 with those published in 2019 (a change in direction of research compared to recent past). We employ 4 different strategies: T1, T2, Y1 and Y2 (see Fig. 1).

#### 2.6.1 Strategy T1: Novel paper detection based on semantic distance

We first analyzed the distribution of the pair-wise distance between all titles in embedded space for the COVID corpus and examined pairs sorted by distance to determine suitable thresholds for relatedness S-rel and, conversely, S-unrel for being semantically unrelated with high probability. There is a grey area in between the two thresholds where pairs of titles at the same distance from each other are sometimes related, and sometimes not. We confirmed the consistency of the thresholds by repeating the calibration check with the leprosy dataset. To determine if a title T is novel relative to a comparator collection C (typically in a preceding time window), we first determine the minimum Euclidean distance E-min between T and all titles in C. All titles T with E-min values higher than S-rel are potentially novel (e.g., the green square in Fig. 1 labeled as T1).

#### 2.6.2 Strategy T2: Detection of novel papers that may constitute a trend

This builds on strategy T1 by requiring that papers not only be distant from an ‘old’ neighborhood (subcollection or time window) but also be part of a ‘trendy’ neighborhood. In other words, a title T is considered to be part of a trend if it lies in a location that corresponds to low-density in the old neighborhood (blue dots) and high-density in the new neighborhood (green squares). This is quantified by requiring that a novel title T that is part of a trend be closer to *k* papers in the new neighborhood (e.g., green squares labeled T2 in Fig. 1) than *k* papers in the old neighborhood. The default value of *k* is set to 3 to correspond to an emerging trend but may be set higher if desired.

#### 2.6.3 Strategy Y1: Detection of a group of novel papers based on their mean vector

Strategy Y1 tracks the location of the mean vector of papers in each time window. Long hops in embedded space may imply a substantial difference in the direction of research (e.g., see Y1 in Fig. 1). The underlying signal may be uncovered by word frequency analysis or titles in high density areas close to the mean.

#### 2.6.4 Strategy Y2: Proportion of novel papers

The premise is that the novelty of a time window (or subcollection of papers) may be gauged by estimating what proportion of papers in that window are at least distance S-rel from all papers in the past. When examining successive time windows, large upward oscillations of this proportion may signal the presence of a new trend.

## 3. Results and Discussion

We use two different approaches to detect changes over time in the focus of research papers on a particular topic. The first approach consists of using changepoint analysis to detect changes in the frequency of words within titles or abstracts. The second approach consists of embedding titles in vector space and using the distance between titles as an approximation of the corresponding semantic difference. To illustrate these approaches, we have chosen to focus on a pair of contrasting infectious diseases, COVID-19 and leprosy. COVID-19 has a short history as a pandemic affecting millions of people, while leprosy is one of the oldest human diseases. While much progress has been made, and effective treatment is available when diagnosed early, around 200,000 new cases continue to be reported each year.^17^

**Figure 2.**
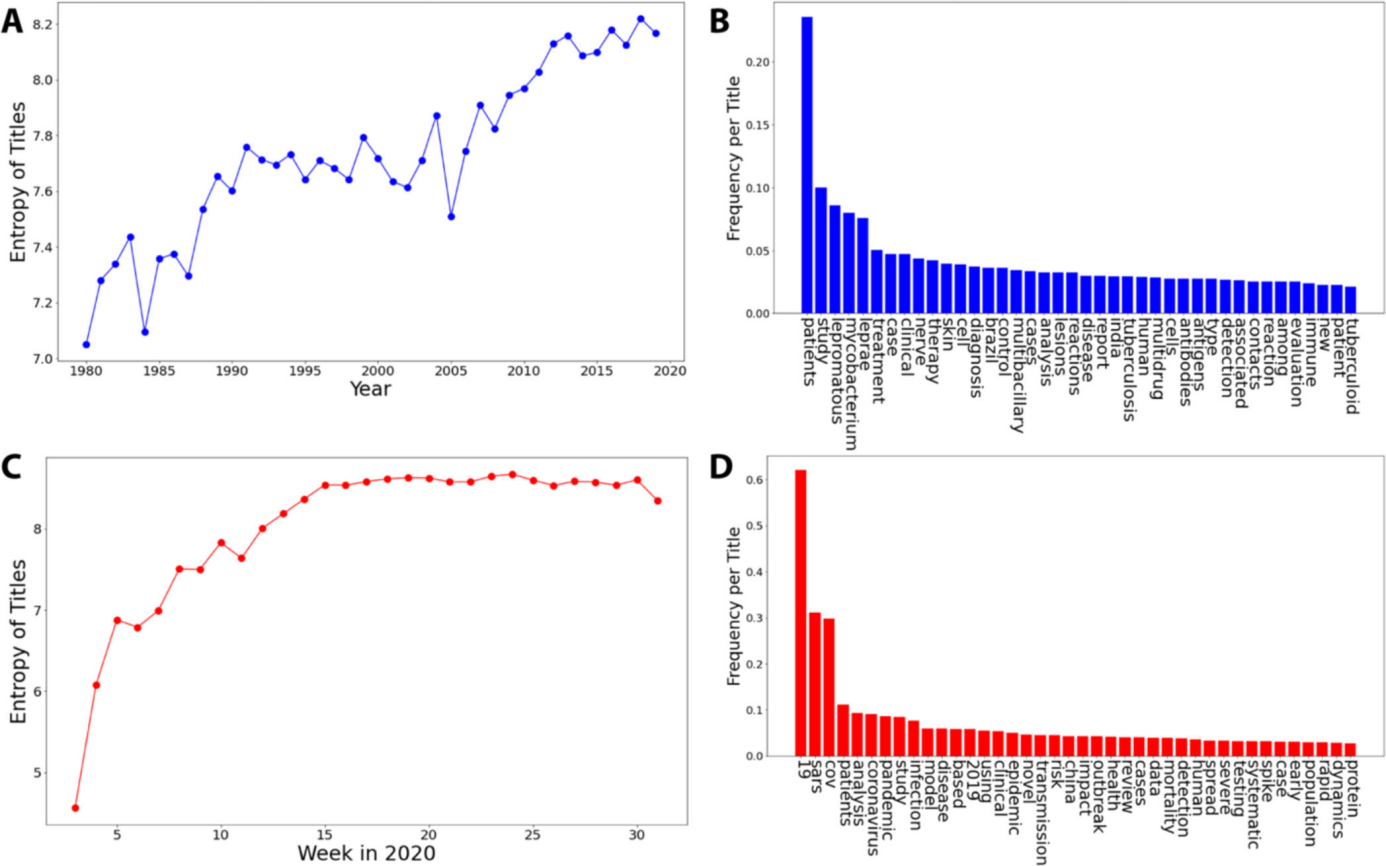
A) Entropy of titles on leprosy. B) Frequency of words in titles on leprosy. C) Entropy of titles on COVID-19. D) Frequency of words in titles on COVID-19.

Fig. 2 shows the increasing complexity of the information content of titles over time, corresponding to the diversity of terms shown on the right. Though the entropy of titles on leprosy has risen over a longer period of time than for COVID, it is interesting that both show a maximum value of just over 8 bits. This suggests that titles of research papers on diseases might have a similar complexity, and by extension share properties of similarity in the embedding space. The most frequent terms occurring within the titles of papers (Fig. 2B and 2D) are used for subsequent changepoint analysis.

**Figure 3.**
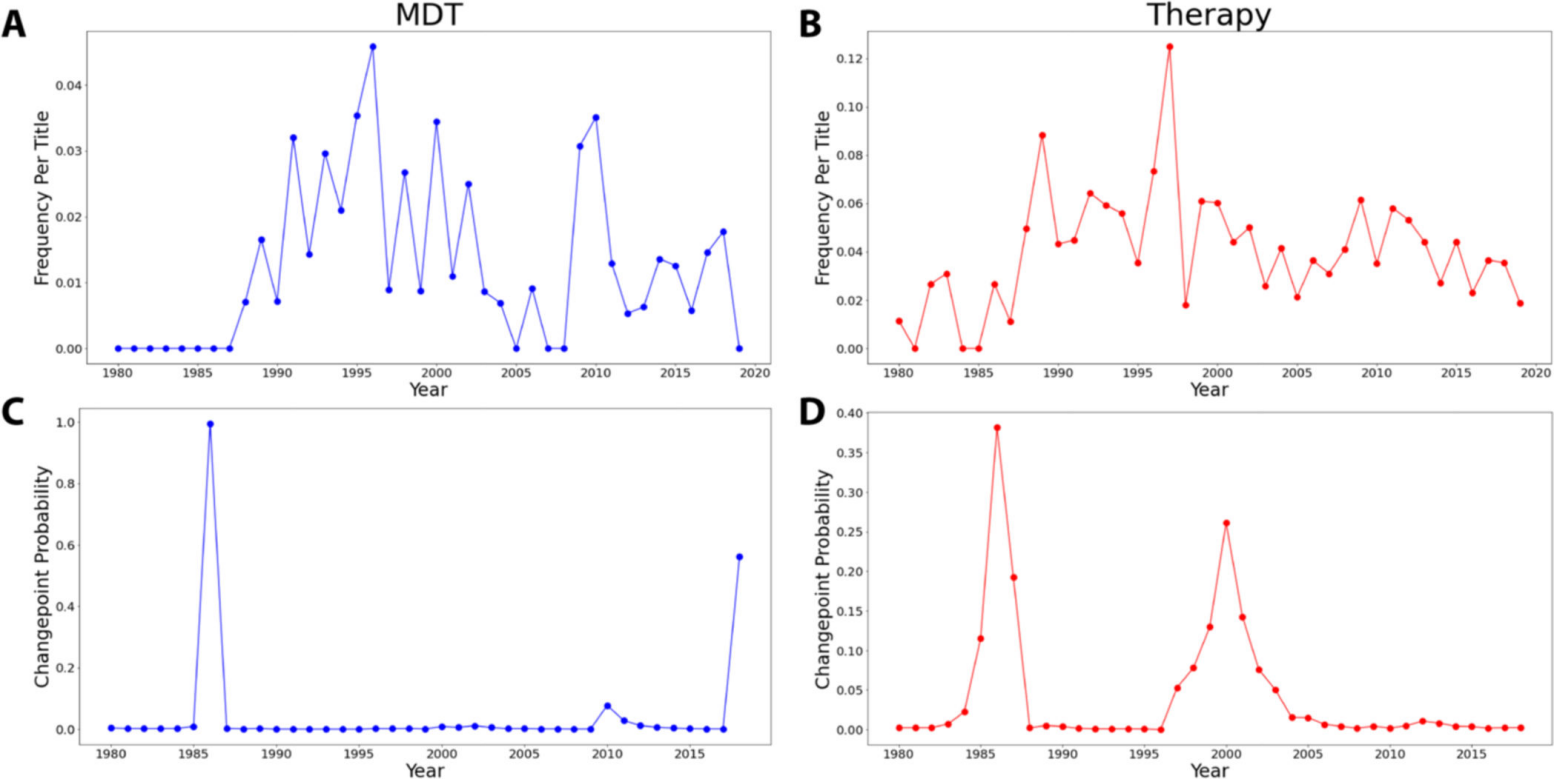
Examples of changepoint analysis of the frequency of the words “MDT” and “Therapy” in titles containing the word “leprosy.” A) Temporal frequency of “MDT.” B) Temporal frequency of “Therapy.” C) Changepoint peaks mark the beginning and end of a period of relatively high frequency of “MDT.” D) Changepoint peaks mark the beginning and end of a period of relatively high frequency of “Therapy.”

**Figure 4.**
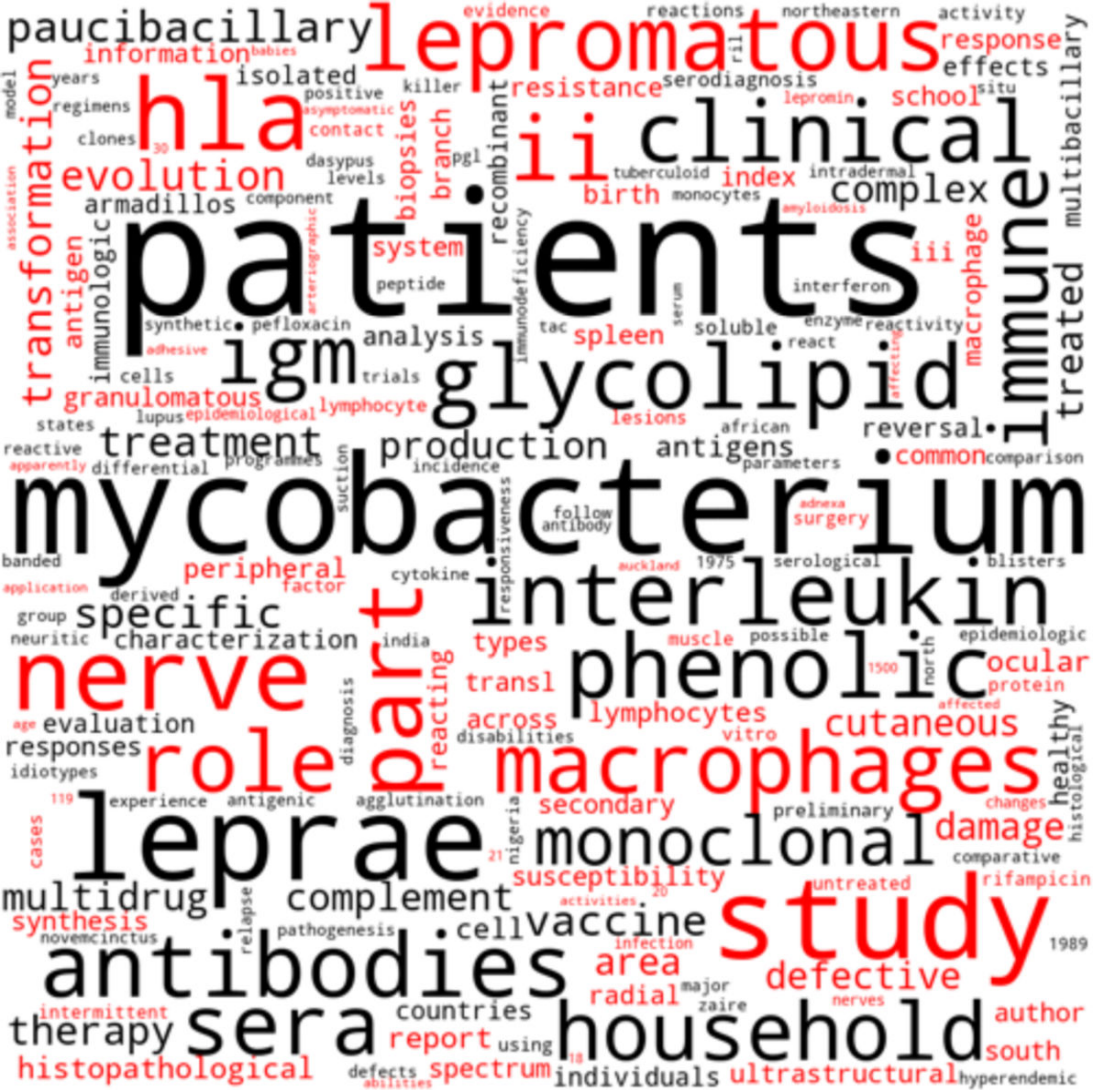
Differential cloud of terms within titles containing the word “leprosy” between the years 1980 and 1990, corresponding to the first changepoint in Fig. 3. Note “multidrug” and “therapy” in lower left corner in similar font size. Black = increase in frequency, Red = decrease in frequency.

Fig. 3 shows the temporal frequencies of some of the most frequent terms occurring within the titles of research papers. The corresponding changepoint analysis highlights points in time when there is a high probability of a significant change in the probability of the occurrence of a term in a title. The following inferences can be made from this figure. First, despite the fluctuations shown in panels A and B, changepoint analysis finds identical changepoints for “MDT” and “Therapy,” indicating a surge of literature mentioning multidrug therapy for the treatment of leprosy. In fact, this corresponds to the period of excitement when (dapsone+rifampin+clofazimine) was recommended by WHO in the 1980s as curative treatment, resulting in the elimination of leprosy as a global public health problem (defined as an incidence of less than 1 in 10000) by the year 2000.^17^ Fig. 4 shows the relative rise and relative fall in the frequency of words spanning a period suggested by the location of changepoints for the terms in Fig. 3. As expected, terms such as the following show an increase in frequency: (multidrug, therapy, treated, patients, paucibacillary, multibacillary) - presumably indicating the advent of successful multidrug therapy. In contrast, terms such as (lepromatous, nerve, cases, granulomatous) show a decrease, presumably corresponding to a decrease in the incidence and morbidity of the disease.

The use of term frequencies as the basis of finding temporal changes in the focus of research papers has the following limitations:

1. While considering terms independently might work well for categories such as drug names, it has limited ability to exploit the meaning of the entire title.
2. Focusing only on the higher frequency terms may miss the true harbingers of change; the long tail of low frequency terms makes it harder to find the significant ones.

Ideally, we would like to project the key focus of research papers into a shared semantic space, without having to count frequencies first. This would make it possible to highlight newly populated regions of the space as containing papers representing new directions. Several models have been published for projecting words into vector spaces^18,19,20^. More recently, it has been shown that models that directly embed a sentence are more accurate than taking the average of the constituent word vectors. Titles of papers typically encode more meaning than a set of words but often fail to form a well-formed sentence. In order to confirm if title embeddings retain inter-title similarity in the same manner as inter-sentence similarity^21^, we used BioSentVec^16^ to embed the titles from the bioRxiv COVID dataset and sorted pairs of titles by increasing Euclidean distance between the corresponding vectors. Representative pairs of related titles are shown in Table 1. Titles may be addressing the same objective except for minor differences in methodology or disease population. As hoped for, the embedded vectors also capture similarity based on implicit meaning. For example, associations are captured between heart injury and raised blood levels of BNP, and between kidney injury and hypertension (supplementary information). Table 2 shows a sample of titles that are unrelated, and further apart in vector space. An empirical threshold of 2.3 corresponds to an FDR of less than 1% for semantic relatedness, and distances above 4 are very unlikely to indicate relatedness (supplementary information for full table).

Based on the embedded space of titles, we used the following ways to identify titles that might be novel compared to the past:

T1. Find titles that are furthest away from any title in the past (Table 3).

T2. Find titles whose location corresponds to a low density area of the set of past titles but high density area in the current time period.

Strategy T1 is aimed at the identification of one of the earliest papers in a possibly new area. Each row in Table 3 lists the paper whose title was the most dissimilar to all previous titles (note the large distances in the right column from the closest title among prior papers). Most of the titles are compatible with being one of the first papers on the topic, with the embedding also highlighting subtle novelties like different types of phylogenetic research. Note that “COVID-19 spreading: a model” could be considered similar to “Spatio-temporal propagation of COVID-19 pandemics,” even though there is minimal lexical overlap. Strategy T2 is aimed at the identification of a burgeoning area compared to the past since it is meant to detect several new titles that are related; it also minimizes the chance of false positives that might confound the results of strategy T1. Examples of titles yielded by strategy T2 are a surge of publications on multidrug therapy in the period 1988–1991 compared to the period until 1987 (3-neighborhood ratio of old:new of 1.35). Examples are:

Leprosy control through multidrug therapy (MDT).

Experience with WHO-recommended multidrug therapy (MDT) for multibacillary (MB) leprosy patients in the leprosy control program of the All Africa Leprosy and Rehabilitation Training Center in Ethiopia: appraisal of the recommended duration of MDT for MB patients.

Bacillaemia in leprosy and effect of multidrug therapy.

A search of PubMed confirms this trend in that the first hit for the query “leprosy AND MDT” is only in 1985.

Based on the embedded space of titles, we used the following ways to identify years that might be novel compared to the past:

### Strategy Y1.

Find the mean title vector for each year, and trace the path from year to year. The longer paths may represent a significant change between adjacent years.

### Strategy Y2.

Estimate the proportion of titles in a given year that are located far away from any title in the preceding time period. A large change in the proportion of such titles in a given year may suggest a new and growing area of research.

**Figure 5.**
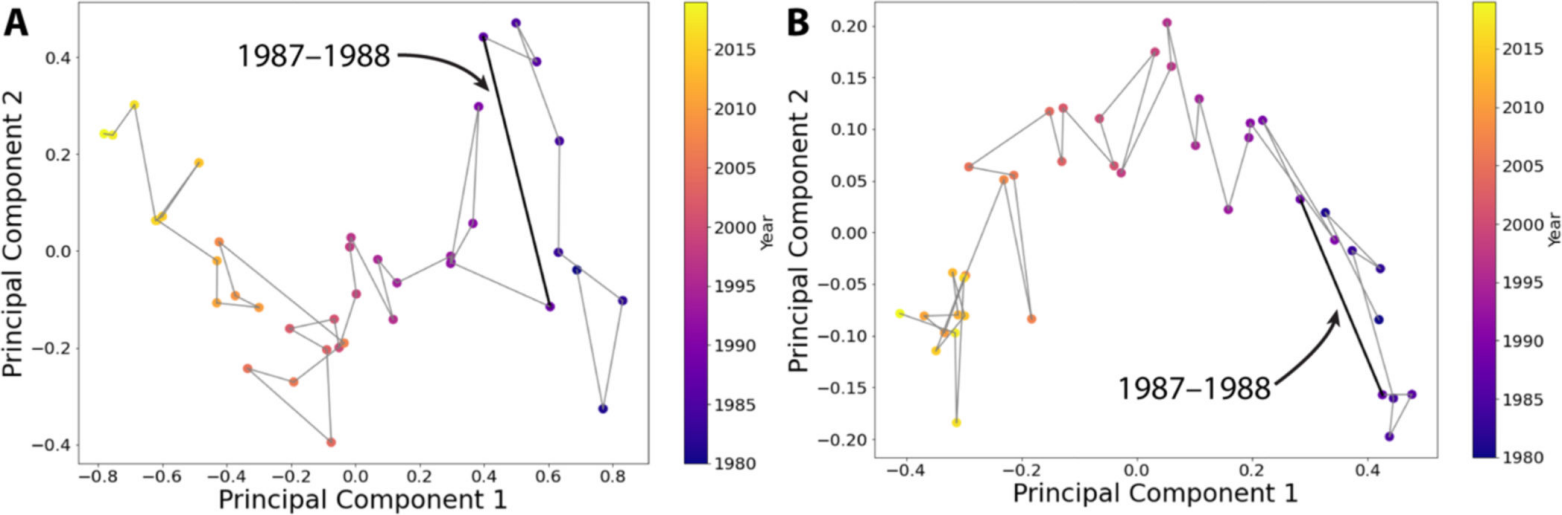
Depiction of strategy Y1: PCA projection of the mean embedded vector from papers on leprosy. Left: embedded titles; Right: embedded abstracts.

**Figure 6.**
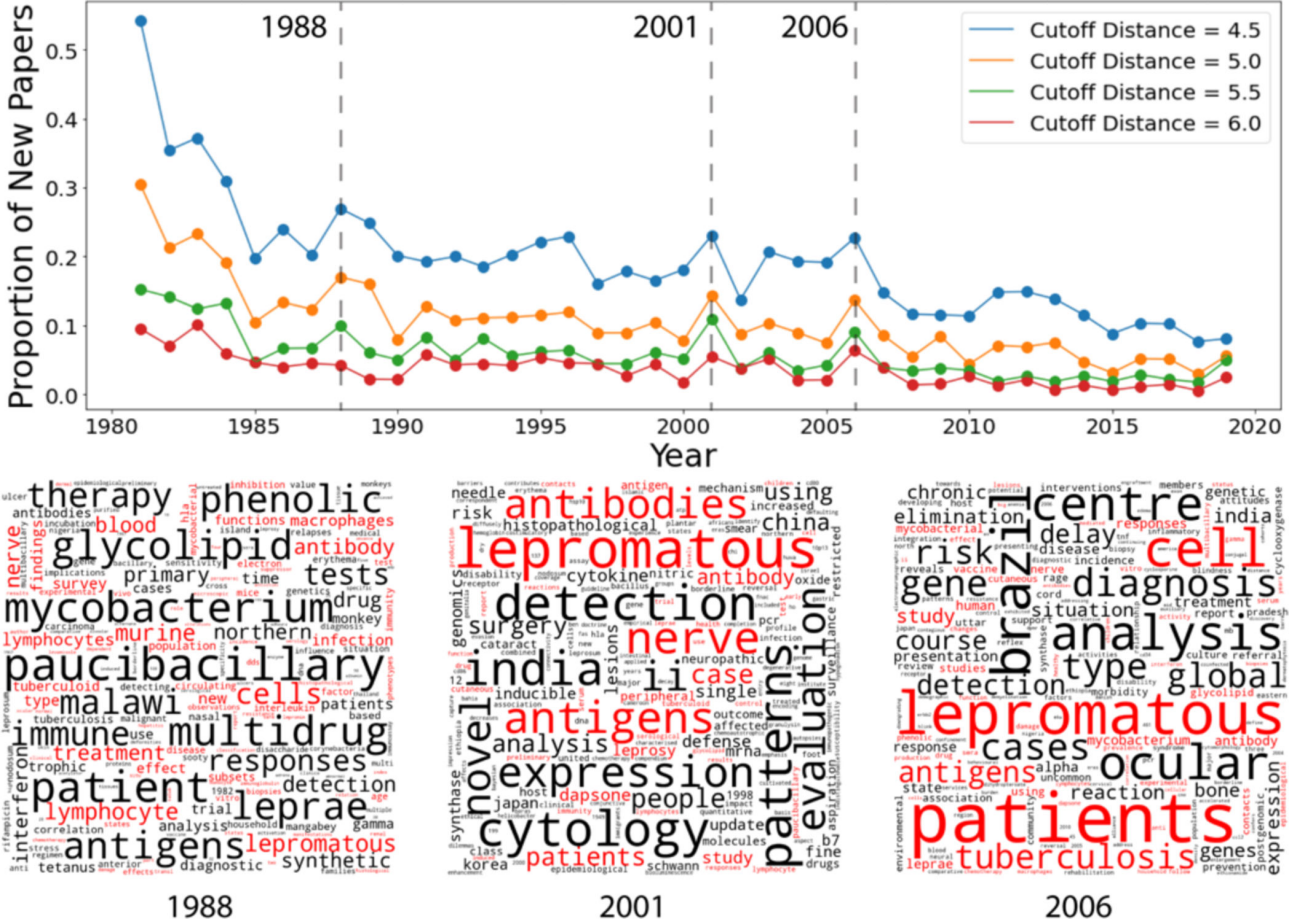
Top: Strategy Y2 depicts the proportion of ‘novel’ papers each year compared to all past publications. Bottom: Differential word clouds of terms within titles containing the word “leprosy” at times detected by Y2.

A low-dimensional projection of strategy Y1 is shown in Figure 5. The long path between 1987 and 1988 is in alignment with previous results (changepoint analysis, Strategy T2) in this paper regarding the literature on multidrug therapy in leprosy. Based on strategy Y2, Fig. 6 shows the proportion of potentially novel titles at each time-point, and corresponding differential word clouds to indicate terms possibly indicative of new areas.

While the title might be the most succinct ‘sentence’ representation of the topic of a paper, a rhetorical or terse title may fail to convey the essence of a paper. We therefore attempted to embed entire abstracts as an alternative version of strategy Y1. While this shows a possibly less noisy path from year to year, the variance (range of values on axes) decreases so that the semantic ‘hops’ from year to year become smaller (Fig. 5). Results from strategies such as Figure 5 and 6 could be used to calibrate and determine the thresholds for measures of novelty (projected path lengths or significant proportion of novel papers) to be indicative of novelty in the recent past.

## 4. Conclusions

We have presented and illustrated approaches to the detection of semantic changepoints within a set of research publications. Admittedly, a novel paper is not synonymous with a high impact paper. Nor is it synonymous with a novel conclusion. False negatives are also possible. For instance, analogous sentences differing in only one term (e.g., a highly effective new therapeutic intervention instead of an older marginally effective one) may have similar embeddings, especially within long titles. More specialized embedding schemes that are domain and problem specific may be necessary. Changepoint analysis is based on acute differences, and therefore less able to detect steady growth in a new area over a longer time period. As proof of concept, we have focused on a subset of the publications for each disease. For detection of truly novel papers, multiple databases will need to be considered together with sophisticated ontology and machine-based querying to maximize recall without sacrificing precision. The approach can be potentially enhanced by using named entity recognition approaches for more insightful analysis of the underlying reasons for an observed changepoint. A more detailed modeling of content based on incorporating topic modeling and/or the analysis of full text journal papers can help provide more granular changepoints and corresponding interpretations. Ultimately, the approach presented in this paper could potentially be incorporated into a real time system for the detection of novel information as it appears. Clinicians could potentially use it to find new vistas in their specific disciplines, especially in the context of hitherto incurable diseases. Researchers could find nascent topics worth expanding into.

## Supplementary Material

Supp-table

## Figures and Tables

**Table 1.** Examples of related titles within the bioRxiv set of papers on COVID.

Title 1	Title 2 (Related)	L2 distance
Predicting the number of reported and unreported cases for the COVID-19 epidemics in China, South Korea, Italy, France, Germany and United Kingdom	Predicting the number of reported and unreported cases for the COVID-19 epidemic in South Korea, Italy, France and Germany	1.429
The impact of current and future control measures on the spread of COVID-19 in Germany	A first study on the impact of current and future control measures on the spread of COVID-19 in Germany	1.844
Characterization of a novel, low-cost, scalable ozone gas system for sterilization of N95 respirators and other COVID-19 use cases	Characterization of a novel, low-cost, scalable vaporized hydrogen peroxide system for sterilization of N95 respirators and other COVID-19 personal protective equipment	2.135
A 5-min RNA preparation method for COVID-19 detection with RT-qPCR	A simple RNA preparation method for SARS-CoV2 detection by RT-qPCR,	2.229
Clinical features and outcomes of 2019 novel coronavirus-infected patients with high plasma BNP levels	Clinical features and outcomes of 2019 novel coronavirus-infected patients with cardiac injury	2.241
Clinical characteristics of Coronavirus Disease 2019 (COVID-19) patients in Kuwait	Clinical and epidemiological characteristics of Coronavirus Disease 2019 (COVID-19) patients	2.318

**Table 2.** Examples of unrelated titles within the bioRxiv set of papers on COVID.

Title 1	Title 2 (Unrelated)	L2 distance
Early Prediction of Disease Progression in 2019 Novel Coronavirus Pneumonia Patients Outside Wuhan with CT and Clinical Characteristics	Epidemiological and Clinical Characteristics of 17 Hospitalized Patients with 2019 Novel Coronavirus Infections Outside Wuhan, China	2.337
Preliminary epidemiological analysis on children and adolescents with novel coronavirus disease 2019 outside Hubei Province in China: an observational study utilizing crowdsourced data	Evolving epidemiology of novel coronavirus diseases 2019 and possible interruption of local transmission outside Hubei Province in China: a descriptive and modeling study	2.351
Clinical course and potential predicting factors of pneumonia of adult patients with coronavirus disease 2019 (COVID-19): A retrospective observational analysis of 193 confirmed cases in Thailand	History of Coronary Heart Disease Increases the Mortality Rate of Coronavirus Disease 2019 (COVID-19) Patients: A Nested Case-Control Study Based on Publicly Reported Confirmed Cases in Mainland China	2.419
The First Consecutive 5000 Patients with Corona virus Disease 2019 from Qatar; a Nation-wide C ohort Study	Knowledge and perceptions of coronavirus disease 2019 among the general public in the United States and the United Kingdom: A cross-sectional online s urvey	3.294
Analysis of hospitalized COVID-19 patients in t he Mount Sinai Health System using electronic medical records (EMR) reveals important progno stic factors for improved clinical outcomes	Core warming of coronavirus disease 2019 (COVID −19) patients undergoing mechanical ventilation: pr otocol for a randomized controlled pilot study	3.663

**Table 3.** Most novel paper each week compared to all previous weeks, as predicted by strategy T1

Week (2020)	Title (farthest from all prior titles on COVID in bioRxiv dataset)	Distance
4	From SARS-CoV to Wuhan 2019-nCoV: Will History Repeat Itself?	6.399
5	Nucleotide Analogues as Inhibitors of Viral Polymerases	7.016
6	Phylogenomic analysis of the 2019-nCoV coronavirus	6.938
7	Transmission Dynamics of 2019-nCoV in Malaysia	7.127
8	Fractal kinetics of COVID-19 pandemic	8.479
9	Application and optimization of RT-PCR in diagnosis of SARS-CoV-2 infection	5.473
10	Mutations, Recombination and Insertion in the Evolution of 2019-nCoV	7.150
11	The architecture of SARS-CoV-2 transcriptome	9.577
12	Routes for COVID-19 importation in Brazil	7.624
13	Spatio-temporal propagation of COVID-19 pandemics	7.462
14	Presence of SARS-Coronavirus-2 in sewage	10.080
15	Work-related Covid-19 transmission	9.856
16	COVID-19 is an emergent disease of aging	6.918
17	Identification of super-transmitters of SARS-CoV-2	16.268
18	*COVID-19 spreading: a model	8.260
19	AI334 and AQ806 antibodies recognize the spike S protein from SARS-CoV-2 by ELISA	9.338
20	Placental pathology in COVID-19	8.308
21	Metamorphosis of COVID-19 Pandemic	11.073
22	Are we #stayinghome to Flatten the Curve?	8.388
23	Cytokine biomarkers of COVID-19	9.935
24	Stability of SARS-CoV-2 Phylogenies	12.348
25	Hypokalemia in Patients with COVID-19	8.513
26	Surveillance testing of SARS-CoV-2	8.335
27	From predictions to prescriptions: A data-driven response to COVID-19	10.656
28	Are men dying more than women by COVID-19?	7.455
29	Cold sensitivity of the SARS-CoV-2 spike ectodomain	11.843
30	Phylogeny of the COVID-19 Virus SARS-CoV-2 by Compression	7.128
31	CAR Macrophages for SARS-CoV-2 Immunotherapy	8.481

* False positive

## References

[1] Blei DM, Ng AY & Jordan MI Latent dirichlet allocation. J. Mach. Learn. Res 3, 993–1022 (2003).

[2] Boyack KW Clustering more than two million biomedical publications: Comparing the accuracies of nine text-based similarity approaches. PloS One 6, e18029 (2011). 10.1371/journal.pone.0018029 PMC3060097 21437291

[3] Chen C. CiteSpace II: Detecting and visualizing emerging trends and transient patterns in scientific literature. J. Am. Soc. Inf. Sci. Technol 57, 359–377 (2006).

[4] Jun S-P, Yoo HS & Choi S. Ten years of research change using Google Trends: From the perspective of big data utilizations and applications. Technol. Forecast. Soc. Change 130, 69–87 (2018).

[5] Batagelj V. Efficient algorithms for citation network analysis. ArXiv Prepr. Cs0309023 (2003).

[6] Wiersinga WJ, Rhodes A, Cheng AC, Peacock SJ & Prescott HC Pathophysiology, Transmission, Diagnosis, and Treatment of Coronavirus Disease 2019 (COVID-19): A Review. JAMA (2020) doi:10.1001/jama.2020.12839. 32648899

[7] WHO | Global Leprosy Strategy 2016–2020: Accelerating towards a leprosy-free world. WHO http://www.who.int/lep/resources/9789290225096/en/.

[8] bioRxiv COVID-19 SARS-CoV-2 preprints from medRxiv and bioRxiv. https://connect.bioRxiv.org/relate/content/181.

[9] Hunter JD Matplotlib: A 2D Graphics Environment. Comput. Sci. Eng 9, 90–95 (2007).

[10] Pedregosa F. Scikit-learn: Machine Learning in Python. J. Mach. Learn. Res 12, 2825–2830 (2011).

[11] Fearnhead P. Exact and efficient Bayesian inference for multiple changepoint problems.(2006).

[12] Adams RP & MacKay DJC Bayesian Online Changepoint Detection. ArXiv07103742 Stat (2007).

[13] Xuan X. & Murphy K. Modeling changing dependency structure in multivariate time series.in Proceedings of the 24th international conference on Machine learning 1055–1062 (Association for Computing Machinery, 2007). doi:10.1145/1273496.1273629.

[14] Bird S, Klein E. & Loper E. Natural language processing with Python. (O’Reilly, 2009).

[15] Mueller A. Python Word Cloud Package. (2020).

[16] Chen Q, Peng Y. & Lu Z. BioSentVec: creating sentence embeddings for biomedical texts. in 2019 IEEE International Conference on Healthcare Informatics (ICHI) 1–5 (2019). doi:10.1109/ICHI.2019.8904728.

[17] WHO fact sheet on leprosy. https://www.who.int/news-room/fact-sheets/detail/leprosy.

[18] Pennington J, Socher R. & Manning CD Glove: Global vectors for word representation. In Proceedings of the 2014 conference on empirical methods in natural language processing (EMNLP) 1532–1543 (2014).

[19] Mikolov T, Chen K, Corrado G. & Dean J. Efficient Estimation of Word Representations in Vector Space. ArXiv13013781 Cs (2013).

[20] Zhang Y, Chen Q, Yang Z, Lin H. & Lu Z. BioWordVec, improving biomedical word embeddings with subword information and MeSH. Sci. Data 6, 52 (2019). 31076572 10.1038/s41597-019-0055-0 PMC6510737

[21] Allot A. LitSense: making sense of biomedical literature at sentence level. Nucleic Acids Res. 47, W594–W599 (2019). 31020319 10.1093/nar/gkz289 PMC6602490

