# Supplementary material for: Semantic Changepoint Detection for Finding Potentially Novel Research Publications
Source: Pac Symp Biocomput. Author manuscript; Available in PMC 2021 Aug 9. (PMC8352552)
Supplement: Supp-table [file NIHMS1709414-supplement-Supp-table.pdf]

Table S1. Examples of related titles within the BioRxiv set of papers on COVID-1*9*

Title 1	Title 2 (Related)	Minor difference	L2 distance
Predicting the number of reported and unreported cases for the COVID-19 epidemics in China, South Korea, Italy, France, Germany and United Kingdom	Predicting the number of reported and unreported cases for the COVID-19 epidemic in South Korea, Italy, France and Germany	Geography	1.429
The impact of current and future control measures on the spread of COVID-19 in Germany	A first study on the impact of current and future control measures on the spread of COVID-19 in Germany	Temporal iteration	1.844
Comparative efficacy and safety of pharmacological interventions for the treatment of COVID-19: A systematic review and network meta-analysis of confounder-adjusted 20212 hospitalized patients	Comparative Efficacy and Safety of Pharmacological Managements for Hospitalized COVID-19 Patients: Protocol for Systematic Review and Trade-Off Network Meta-Analysis	Methodology	2.103
Characterization of a novel, low-cost, scalable ozone gas system for sterilization of N95 respirators and other COVID-19 use cases	Characterization of a novel, low-cost, scalable vaporized hydrogen peroxide system for sterilization of N95 respirators and other COVID-19 personal protective equipment	Chemical	2.135
Hypertension in patients hospitalized with COVID-19 in Wuhan, China: A single-center retrospective observational study	Acute kidney injury in patients hospitalized with COVID-19 in Wuhan, China: A single-center retrospective observational study	Pathology	2.156
Clinical Characteristics of Recurrent-positive Coronavirus Disease 2019 after Curative Discharge: a retrospective analysis of 15 cases in Wuhan China	Epidemiological and clinical features of 291 cases with coronavirus disease 2019 in areas adjacent to Hubei, China: a double-center observational study	Disease population	2.176
Protocol for a randomized controlled trial testing inhaled nitric oxide therapy in spontaneously breathing patients with COVID-19	Protocol of a randomized controlled trial testing inhaled Nitric Oxide in mechanically ventilated patients with severe acute respiratory syndrome in COVID-19 (SARS-CoV-2)	Administration mode	2.215
A 5-min RNA preparation method for COVID-19 detection with RT-qPCR	A simple RNA preparation method for SARS-CoV-2 detection by RT-qPCR,	None	2.229
Clinical features and outcomes of 2019 novel coronavirus-infected patients with high plasma BNP levels	Clinical features and outcomes of 2019 novel coronavirus-infected patients with cardiac injury	Pathology	2.241
Clinical characteristics of Coronavirus Disease 2019 (COVID-19) patients in Kuwait	Clinical and epidemiological characteristics of Coronavirus Disease 2019 (COVID-19) patients	Geography, Aspect	2.318

Table S2. Examples of unrelated titles within the BioRxiv set of papers on COVID-19

Title 1	Title 2 (Unrelated)	L2 distance
Early Prediction of Disease Progression in 2019 Novel Coronavirus Pneumonia Patients Outside Wuhan with CT and Clinical Characteristics	Epidemiological and Clinical Characteristics of 17 Hospitalized Patients with 2019 Novel Coronavirus Infections Outside Wuhan, China	2.337
Preliminary epidemiological analysis on children and adolescents with novel coronavirus disease 2019 outside Hubei Province in China: an observational study utilizing crowdsourced data	Evolving epidemiology of novel coronavirus diseases 2019 and possible interruption of local transmission outside Hubei Province in China: a descriptive and modeling study	2.351
Clinical course and potential predicting factors of pneumonia of adult patients with coronavirus disease 2019 (COVID-19): A retrospective observational analysis of 193 confirmed cases in Thailand	Epidemiological and clinical features of 291 cases with coronavirus disease 2019 in areas adjacent to Hubei, China: a double-center observational study	2.351
Clinical course and potential predicting factors of pneumonia of adult patients with coronavirus disease 2019 (COVID-19): A retrospective observational analysis of 193 confirmed cases in Thailand	History of Coronary Heart Disease Increases the Mortality Rate of Coronavirus Disease 2019 (COVID-19) Patients: A Nested Case-Control Study Based on Publicly Reported Confirmed Cases in Mainland China	2.419
The First Consecutive 5000 Patients with Coron avirus Disease 2019 from Qatar; a Nation-wide Cohort Study	Knowledge and perceptions of coronavirus diseas e 2019 among the general public in the United Sta tes and the United Kingdom: A cross-sectional onl ine survey	3.294
Second week methyl-prednisolone pulses impro ve prognosis in patients with severe coronaviru s disease 2019 pneumonia: an observational co mparative study using routine care data	Estimating the proportion of coronavirus disease 2019 (COVID-19) cases among households in Fran ce : a cross-sectional study on individuals with my ocardial infarction history	3.294
Analysis of hospitalized COVID-19 patients in t he Mount Sinai Health System using electronic medical records (EMR) reveals important progn ostic factors for improved clinical outcomes	Core warming of coronavirus disease 2019 (COVI D-19) patients undergoing mechanical ventilation: protocol for a randomized controlled pilot study	3.663
Impact Assessment of Full and Partial Stay-at-H ome Orders, Face Mask Usage, and Contact Tra cing: An Agent-Based Simulation Study of CO VID-19 for an Urban Region	Epidemiological investigation of the first 135 CO VID-19 cases in Brunei: Implications for surveilla nce, control, and travel restrictions	3.663
